# Lynch syndrome mutation spectrum in New South Wales, Australia, including 55 novel mutations

**DOI:** 10.1002/mgg3.198

**Published:** 2016-01-11

**Authors:** Wenche Sjursen, Mary McPhillips, Rodney J. Scott, Bente A. Talseth‐Palmer

**Affiliations:** ^1^ Department of Laboratory MedicineChildren's and Women's HealthFaculty of MedicineNorwegian University of Science and Technology7491TrondheimNorway; ^2^ Department of Pathology and Medical GeneticsSt. Olavs HospitalTrondheim University Hospital7006TrondheimNorway; ^3^ Division of Molecular MedicinePathology NorthNewcastleNew South WalesAustralia; ^4^ School of Biomedical Sciences and PharmacyFaculty of Health and MedicineUniversity of NewcastleNewcastleNew South WalesAustralia; ^5^ Hunter Medical Research InstituteNewcastleNew South WalesAustralia; ^6^ The Cancer DepartmentMøre og Romsdal Hospital TrustMoldeNorway; ^7^ Department of Research and DevelomentMøre og Romsdal Hospital TrustMoldeNorway

**Keywords:** Genetic testing, InSiGHT classification, interpretation of mutations, lynch syndrome, mismatch repair genes, pathogen mutations

## Abstract

**Background:**

Lynch syndrome, the most frequent hereditary colorectal cancer syndrome, is caused by defects in mismatch repair genes. Genetic testing is important in order to identify mutation carriers who can benefit from intensive surveillance programs. One of the challenges with genetic testing is the interpretation of pathogenicity of detected DNA variants. The aim of this study was to investigate all putative pathogenic variants tested for at the Division of Molecular Medicine, Pathology North, in Newcastle, Australia, to establish whether previous variant classification is in accordance with that recently performed in the InSiGHT collaboration.

**Methods:**

Prediction programs and available literature were used to classify new variants or variants without classification.

**Results:**

We identified 333 mutation positive families, in which 211 different putative pathogenic mismatch repair mutations were found. Most variants with an InSiGHT classification (141 out of 146) were in accordance with our classification. Five variants were discordant, of which one can definitively be reclassified according to the InSiGHT scheme as class 5. Sixty‐four variants had not been classified by InSiGHT, of whom 55 have not been previously reported.

**Conclusion:**

In conclusion, we found that our classifications were mostly in accordance with the InSiGHT scheme. In addition to already known MMR mutations, we have also presented 55 novel pathogenic or putative pathogenic mutations.

## Introduction

Lynch syndrome (OMIM #120435), formerly called hereditary nonpolyposis colorectal cancer (HNPCC), is the most frequent hereditary predisposition to colorectal cancer (CRC). Lynch syndrome patients also have an increased risk of developing other epithelial malignancies, including endometrial, ovarian, stomach, hepatobiliary, urinary, small bowel, brain, and sebaceous tumors (recently reviewed) (Cohen and Leininger 2014).

Genetic testing is currently offered to families that fulfill the clinical diagnostic criteria as defined by the Amsterdam I and II, or Bethesda guidelines. The four DNA mismatch repair (MMR) genes associated with Lynch syndrome; *MLH1* (OMIM *120436),* MSH2* (OMIM *609309), *MSH6* (OMIM *600678), and *PMS2* (OMIM *600259) are the only ones that are routinely screened in predisposition testing. Recently, germline *EPCAM* (OMIM *185535) deletions have been associated with *MSH2* epimutation, which results in gene expression silencing. Microsatellite instability is the hallmark of Lynch syndrome (Umar et al. 2004) and is detected by size fractionation of a series of mono‐ or di‐nucleotide repeat sequences. Immunohistochemical (IHC) testing of tumor tissue looking for the loss of MMR gene expression is used as a guide for the selection of genes to test.

Lynch syndrome families have an earlier onset of cancer than the general population, and it is therefore imperative to identify high‐risk patients from within these families in order to detect tumors at curable stages. The clinical criteria are useful for the identification of HNPCC, but only approximately half of the identified HNPCC families' harbor a pathogenic MMR mutation that can be reclassified as Lynch syndrome (Sjursen et al. 2010; Steinke et al. 2014). It is therefore important to perform genetic testing in order to identify high‐risk mutation carriers.

One of the challenges with genetic testing is the interpretation of detected DNA variants, which is often straight forward when the variant is a stop codon, a frameshift deletion or insertion, a splice mutation in highly conserved splice donor or acceptor sites (exon intron boundaries), or a gross deletion. All these alterations will alter the protein function, leading to a defective MMR. However, when the alteration is a missense mutation (substitution of amino acid), an in‐frame deletion or insertion, or an intron mutation outside the splice donor or acceptor site, the interpretation can be very complex, requiring extended analyses and even then may not reveal the true nature of the change. Those missense changes that cannot be readily classified are termed variants of uncertain clinical significance (VUS). A 5‐tiered schemes for classification of variants has been made (Plon et al. 2008; Thompson et al. 2013), in which class 1 and 2 variants are not or most likely not pathogenic, respectively, VUS are class 3 variants, whereas class 4 and 5 are likely pathogenic and pathogenic, respectively. The classification of variants is important because class 4 and 5 is used to confirm a Lynch syndrome diagnosis and predictive testing can subsequently be offered to family members. In 2014, the International Society for Gastrointestinal Hereditary Tumours (InSiGHT) developed, tested and applied the 5‐tiered scheme for the classification of constitutional variants in *MLH1*,* MSH2*,* MSH6*, and *PMS2* (Thompson et al. 2014).

The aim of this study was to investigate all putative pathogenic variants tested for at the Division of Genetics, Hunter Area Pathology Service (HAPS), Pathology North, in Newcastle, New South Wales, Australia, to establish whether previous variant classification is in accordance with that performed in the InSiGHT collaboration.

This report is a follow‐up study from Scott et al. (2001) and from Talseth‐Palmer et al. (2010) which presented data from 32 (*MLH1* or *MSH2*) and 35 (*MSH6* or *PMS2*) mutation positive families, respectively. Here, we present data from 333 mutation positive families.

## Material and Methods

The study complies with the requirements of the Hunter New England Human Research Ethics Committee and the University of Newcastle Human Research Ethics Committee, Newcastle, NSW, Australia. Written informed consent was obtained from all participants.

HNPCC probands referred to HAPS (Pathology North) for genetic testing between the years 1997 and 2010 were used in this study. A collection of over 2000 patients diagnosed with HNPCC were included in the study, of which 834 have a molecular diagnosis of Lynch syndrome (germline changes in DNA MMR genes) belonging to 333 families.

Mutation analyses for *MLH1, MSH2,* and *MSH6* were performed at the Division of Genetics, HAPS, Pathology North in Newcastle, New South Wales (NSW), Australia as described in Scott et al. 2001 and Talseth‐Palmer et al. 2010. *PMS2* gene analyses were performed at IMVS Pathology, Adelaide, Australia. Suspected splice variants were further tested by RNA analysis. For RNA analysis, transformed lymphocytes were grown in culture from which RNA was isolated and converted to cDNA, RNA transcripts were size fractionated and any that were not of the expected size were subjected to Sanger sequencing. RNA analyses were performed for the following variants: *MLH1*: c.588+1G>T and c.1731G>A, *MSH2* c.1759G>T and *MSH6*: c.3173‐22_3173‐11del, c.3646+2dupT and c.3556+3_3556+13del.

As all 333 probands harbored a germline MMR gene defect, they were defined as having Lynch syndrome. Members of these families were offered predictive genetic testing.

The Leiden Open Variation Database (LOVD) database (http://chromium.liacs.nl/LOVD2/colon_cancer/variants) was utilized in order to find the variants database ID number and their InSiGHT classifications if reported. This classification system is described in (Thompson et al. 2013). If the variant was not reported in the LOVD database, we used the same system as InSiGHT for classification. Available prediction programs and research literature were also utilized for annotation of pathogenicity, including Alamut software (Interactive Biosoftware, Rouen, France). The following tools and measures were used to assess the functional impact at protein level of observed variants: Grantham's distance (Grantham 1974), PhyloP (Pollard et al. 2010), SIFT (Kumar et al. 2009), MutationTaster (Schwarz et al. 2014), PolyPhen2 (Adzhubei et al. 2010), Mutation Assessor (Reva et al. 2011), NCBI Conserved Domain Database (http://www.ncbi.nlm.nih.gov/cdd/) (Marchler‐Bauer et al. 2015), and UniProt (http://www.uniprot.org/uniprot/). The splice prediction tools used were SpliceSiteFinder‐like (Zhang 1998), MaxEntScan (Yeo and Burge 2004), NNSPLICE (Reese et al. 1997), GeneSplicer (Pertea et al. 2001), and Human Splicing Finder (Desmet et al. 2009). Literature search by PUBMED, ClinVar (Landrum et al. 2014), and Google Scholar were performed to check if the variants had been reported previously.

Reference sequences used in this study are MLH1: NM_000249.2, MSH2: NM_000251.2, MSH6: NM_000179.2, and PMS2: NM_000535.5, and the nomenclature used are as recommended by the Human Gene Mutation Database (HGMD) (Den Dunnen and Antonarakis 2001).

All new MMR variants identified in our cohort, and which are included in this paper are submitted to the LOVD/InSiGHT database.

## Results

A total of 333 mutation positive families were identified; 147 with *MSH2* (44%), 121 with *MLH1* (36%), 57 with *MSH6* (17%), and 8 with *PMS2* (2%) mutations (Table 1). The Amsterdam II criteria were fulfilled for 87 families with *MSH2* (59%), 74 with *MLH1* (61%), 27 with *MSH6* (47%), and 5 with *PMS2* (63%) mutations. In total 834 mutation positive patients were identified, 494 were females and 340 were males.

**Table 1:** Number of mutation positive families and number of variants in each mismatch repair (MMR) gene

Gene	Class 4–5		“Class 3”		Tot no of mut + family members
	No of families	No of variants^a^	No of families	No of variants^a^	
MLH1	119	74 (21)	2	2	329
MSH2	146	90 (27)	1	1	319
MSH6	56	37 (14)	1	1	176
PMS2	7	4 (2)	1	2 (1)	10
Total	328	205 (64)	5	6 (1)	834

a In brackets: variants without InSiGHT classification.

Two hundred and eleven different MMR variants (205 class 4/5 variants and 6 class 3 variants), which were considered to be the cause of Lynch syndrome, were identified in these families (Table 1). Of these variants, 141 were found to have a LOVD DB‐ID number and an InSiGHT classification (class 4 and 5), which were in accordance with our own interpretation (Table S1). Eighty‐four of these variants were found in single families, 29 were found in two families, 13 were found in three families, eight were found in four families, two were found in five families, three were found in six families, whereas one variant was found in eight and eleven families, respectively. The MMR mutation distribution of these 141 variants was 63 variants in *MSH2*, 53 in *MLH1*, 23 in *MSH6*, and 2 in *PMS2*.

In addition, 64 class 4 and 5 variants without any InSiGHT classification were identified and they are presented in Table 2; with 27, 21, 14, and 2 occurring in *MSH2*,* MLH1*,* MSH6,* and *PMS2*, respectively. These variants were interpreted as class 4 or 5 as they were frameshift (*n* = 36), nonsense (*n* = 8), exon deletions or duplications (*n* = 9), splice mutations (*n* = 9), larger in‐frame duplication (*n* = 1) or indel variants leading to stop codon (*n* = 1).

**Table 2:** The 64 variants without any InSiGHT/LOVD classification, which were classified as pathogenic or likely pathogenic in this study (class 4–5)

Gene	Nucleotide change^b^	Consequence of mutation	References or LOVD DB‐ID^a^	Our class
MLH1	c.210_213del	Frameshift, p.(Glu71Ilefs*20)	Scott et al. (2001)	5
	c.333_334delTC	Frameshift, p.(His112Cysfs*9)	not in LOVD	5
	c.420delA	Frameshift, p.(Lys140Asnfs*20)	MLH1_01460^(a)^	5
	c.551C>A	Nonsense, p.(Ser184*)	Not in LOVD	5
	c.582delT	Frameshift, p.(Lys196Asnfs*6)	Not in LOVD	5
	c.662del	Frameshift, p.Gly221Glufs*8	Not in LOVD	5
	c.728_732delATGGT	Frameshift, p.(Asn243Ilefs*62)	Not in LOVD	5
	c.791‐1G>A	Splice site, c.791+1G>T (class 4) and G>C (class 5) is reported	MLH1_01741^(b)^	4
	c.1358dup^(1)^	Frameshift, p.(Thr455Aspfs*24)	Not in LOVD	5
	c.1451del	Frameshift, p.(Asp484Valfs*7)	Not in LOVD	5
	c.1519_1520delTT	Frameshift, p.(Leu507Glufs*7) (c.1520del is reported; class 5)	Not in LOVD	5
	c.1559_2103del^(2)^	Frameshift,p.(Val520Glufs*2)	not in LOVD	5
	c.1656dup	Frameshift, p.(Thr553Hisfs*4)	Not in LOVD	5
	c.1667G>A	Missense, p.(Ser556Asp) Last base in exon (c.1667G>T, MLH1_01162, is reported as class 5)	MLH1_01740^(b)^	4
	c.1668‐2A>C	Splice site, p.?	Not in LOVD	4
	c.1769T>G	Nonsense, p.(Leu590*)	Not in LOVD	5
	c.1834_1837delinsT	Insertion/deletion, p.(Val612*)	Not in LOVD	5
	c.2089dup	Frameshift, p.(Leu697Profs*7)	Not in LOVD	5
	c.2114delC	Frameshift, p.Pro705Leufs*78	Not in LOVD	4
	c.2149delG	Frameshift, p.Glu717Asnfs*66	Not in LOVD	4
	c.2196delA	Frameshift, p.Lys732Asnfs*51	Not in LOVD	4
MSH2	c.1_2458del	Exon deletion, exon 1‐14	Not in LOVD	5
	c.212_792del	Exon deletion, exon 2‐4 (Duplication is reported)	Not in LOVD	5
	c.212del	Frameshift, p.(Gly71Glufs*13)	Not in LOVD	5
	c.366+1G>A	Splice site, p.? (c.366+1G>T is reported as class 4, MSH2_01210)	Not in LOVD	4
	c.367_2805del	Exon deletion, exon 3‐16	Not in LOVD	5
	c.454delA	Frameshift, p.(Met152Cysfs*22)	Not in LOVD	5
	c.478_479delCA	Frameshift, p.(Gln160Glyfs*17)	Not in LOVD	5
	c.647_648dup	Frameshift, p.(Ile217*)	Not in LOVD	5
	c.655dupA	Frameshift, p.(Arg219Lysfs*13)	Not in LOVD	5
	c.793_1386dup	Exon duplication, exon 5‐8 (Frameshift)	Not in LOVD	5
	c.793_2805dup	Exon duplication, exon 5‐16 (not altering reading frame)	Not in LOVD	4
	c.803C>A^(3)^	Nonsense, p.(Ser268*)	Not in LOVD	5
	c.1042C>T	Nonsense, p.(Gln348*)	Not in LOVD	5
	c.1076+1G>C	Splice site, p.? (c.1076+1G>A, and c.1076+1G>T, reported as class 5 and 4, respectively)	Not in LOVD	4
	c.1077_1386del^c^	Exon deletion, exon 7‐8,	Not in LOVD	5
	c.1225C>T	Nonsense, p.(Gln409*)	Not in LOVD	5
	c.1411A>T	Nonsense, p.(Lys471*)	Not in LOVD	5
	c.1510+1G>C	Splice site, p.?	Not in LOVD	4
	c.1536dup	Frameshift, p.(Leu513Thrfs*16)	Not in LOVD	5
	c.1599delT	Frameshift, p.(Arg534Valfs*9)	Not in LOVD	5
	c.1609A>T	Nonsense, p.(Lys537*)	Not in LOVD	5
	c.1662_2634del	Exon deletion, exon 11‐15	Not in LOVD	5
	c.1737dupA	Frameshift, p.(Glu580Argfs*18)	MSH2_01027 Somatic”	5
	c.1759G>T	Splice site, r.1662_1759del, p.(Ser554Argfs*11	Not in LOVD	5
	c.1760‐?_2005+?del	Exon deletion, exon 12, (in‐frame)	Not in LOVD	4
	c.1828_1834delinsGA^(3)^	Frameshift, p.(His610Aspfs*32)	Not in LOVD	5
	c.2051_2116dup^(4)^	In frame duplication of 22 amino acids, p.(Val684_Val705dup)	MSH2_01481	4
MSH6	c.675_676dup	Frameshift, p.(Glu226Valfs*21)	Talseth‐Palmer et al. (2010)	5
	c.900_901insTC	Frameshift, p.(Lys301Serfs*5)	Not in LOVD	5
	c.979del	Frameshift, p.(Thr327Leufs*11)	Not in LOVD	5
	c.1136_1139del	Frameshift, p.(Arg379Metfs*31) (c.1135_1139del, MSH6_00379, is reported as class 5)	Not in LOVD	5
	c.1767del	Frame shift, p.(Pro591Glnfs*19)	Not in LOVD	5
	c.1804_1805delTC	Frameshift, p.(Ser602Lysfs*4)	Not in LOVD	5
	c.3037_3041del	Frameshift, p.(Lys1013Valfs*3)	Not in LOVD	5
	c.3108_3109del	Frameshift, p.(Phe1037Leufs*2)	Not in LOVD	5
	c.3142C>T	Nonsense, p.(Gln1048*)	Not in LOVD	5
	c.3173‐22_3173‐11del	Splice site, r.3173_3438del, p.(Asp1058Glyfs*17)	Talseth‐Palmer et al. (2010)	5
	c.3259_3260insA^(5)^	Frameshift, p.(Pro1087Hisfs*6) (c.3259_3260insT, MSH6_00774, is reported as class 5)	Not in LOVD	5
	c.3560_3563del	Frameshift, p.(Glu1187Valfs*7)	Not in LOVD	5
	c.3573dup	Frameshift, p.(Val1192Cysfs*2)	Not in LOVD	5
	c.3646+2dupT	Splice site, r.3557_3646del p.(Glu1187_Gly1216del)	Not in LOVD	5
PMS2	c.538_903del	Exon deletion, exon 6‐8	(Bakry et al. 2014)	5
	c.746_753del	Frameshift, p.(Asp249Valfs*2)	Talseth‐Palmer et al. (2010)	5

Reference sequences used are MLH1: NM_000249.2, MSH2: NM_000251.2, MSH6: NM_000179.2, and PMS2: NM_000535.5

a Reported by (a) Rodney J. Scott, Newcastle, Australia; (b) James Whitworth, Birmingham, UK.

b Mutation originally identified by: (1) Pathology and Laboratory Medicine, Mount Sinai Hospital,Toronto, Canada; (2) Institute of Medical and Veterinary Science, South Australia; (3) Queensland Health Pathology Service, Molecular Genetics Laboratory, Australia; (4) Victorian Clinical Genetics Service, Molecular Genetics Laboratory; (5) All Wales Laboratory Genetics Service, Institute of Medical Genetics, University Hospital of Wales, Cardiff, UK.

c MSH2 c.1398T>G found in the same patient (VUS).

Five of the nine splice mutations are in the highly conserved splice donor or acceptor sites (±1–2). Three mutations considered to affect splicing (Table 2) has been confirmed by RNA analyses in our laboratory to cause aberrant splicing (pathogenic); two *MSH6* variants (c.3173‐22_3173‐11del and c.3646+2dup) and one *MSH2* variant (c.1759G>T). One predicted splice mutation is located in the last nucleotide of exon 14 (*MLH1* c.1667G>A) and is predicted by three in silico tools to alter splicing.

Sixty of the variants without an InSiGHT classification were found in single families, whereas four variants were found in two families. Only ten of these variants have previously been published, thus fifty‐four of the variants are novel.

Families with only an identified class 1, 2, or 3 MMR variant were not included in this study. But according to the LOVD database five of the variants, which we have interpreted as class 4 or 5, are class 3 based on “insufficient evidence” (Table 3). These are two variants in *MLH1* (c.1A>G and c.988_990del), one in *MSH2* (c.2635‐3C>G)*,* one in *MSH6* (c.3556+3_+13del), and one in *PMS2* (c.1A>G). The start codon *PMS2* variant were found together with a novel (class 3) *PMS2* variant in the same patient (an in‐frame deletion, c.834_842del).

**Table 3:** “Class 3” variants which have been used in predictive testing, that is, they are assumed to be causative pathogenic variants

Gene	Variant	Predicted effect	AMS+	LOVD DB‐ID	Comments
MLH1	c.1A>G	p.Met1? Start loss Alternative transcript	Yes	MLH1_01457 Scott et al. (2001)	Shortened protein with loss of in vitro MMR activity (Parsons et al. 2015)
	c.988_990del	p.Ile330del In frame deletion	Yes	MLH1_01631	Minigene assay: partial exon 11 skipping (Tournier et al. 2008) p.[Ser295Argfs*21, Ile330del]
MSH2	c.2635‐3C>G	Splice mutation	Yes	MSH2_01371	Strong family history
MSH6	c.3556+3_+13del	Splice mutation; p.Ala1147Valfs*9	No	MSH6_00661	Our RNA analysis showed skipping of exon 6
PMS2	c.1A>G^a^	p.Met1? Start loss	No	PMS2_00130	Proband had both CRC and uterine cancer at 55 years
	c.834_842del^a^	In frame deletion, p.His278Gly281 delinsGln		Not previously reported	p.His278_Gly281 is in highly conserved domain

Reference sequences used are MLH1: NM_000249.2, MSH2: NM_000251.2, MSH6: NM_000179.2, and PMS2: NM_000535.5

a Both variants found in the same patient.

*MLH1* c.1A>G (MLH1_0001457) has been reported once in the LOVD database by us, and *PMS2* c.1A>G has been reported six times to the LOVD database. They are both classified as VUS by InSiGHT due to insufficient evidence. The in‐frame deletion in *MLH1*, c.988_990del, p.(Ile330del) (MLH1_01631) has been reported in four patients in the LOVD database. We argue for the pathogenicity of these three variants in the discussion.

The predicted splice mutation in *MSH6* c.3556+3_+13del (MSH6_00661) has been reported twice to the LOVD database and it is classified as a VUS due to the absence of RNA analyses. RNA analysis was performed in our laboratory showing that the mutation results in skipping of exon 6. Another predicted splice mutation in *MSH2* c.2635‐3C>G (MSH2_01371) has been reported twice to the LOVD database and was classified as a VUS due to the lack of RNA analysis. RNA analysis was not available for this study either. Three in silico splice prediction tools predicted this variant to alter the acceptor site 3 bps downstream by a change in −70.8% compared to wild type (WT). Our family harboring this mutation has a strong family history of cancer; five family members were found to be mutation carriers, three with CRC (diagnosed at 26, 35, and 49 years of age), one with adenomas at 26 years of age and one unaffected in his twenties. Thus, the *MSH2* c.2635‐3C>G mutation may be a high‐ risk mutation in this family, although cDNA analysis needs to be performed before a conclusion can be made.

The class 3 *PMS2* variant (c.834_842del) found together with *PMS2* c.1A>G in one of our patient has not been reported previously. This deletion causes the loss of 4 residues in exon 8 (His278, Gly279, Val280, and Gly281) and the insertion of Gln. His278, Gly279, and Gly281 are highly conserved residues in protein domain MutL_Trans_hPMS_2_like, thus, they are likely important for correct function (transducer domain, important in the transduction of structural signals from ATP‐binding site to the DNA breakage/reunion regions of the enzymes). Structurally, residue p.278‐280 form a turn between two beta‐strands (http://www.uniprot.org/uniprot/P54278). Therefore, it is possible that the observed deletion introduces a globular change in the protein structure, leading to altered protein function.

An overview of the distribution of MMR mutation types of the 211 MMR variants reported in this study is shown in Table 4. Frameshift (86/41%), splice site (43/20%), nonsense (37/18%), and exon deletion/duplication (31/15%) mutations were the most frequent mutation types.

**Table 4:** Overview of the distribution of all the different MMR mutation types reported in this study (including data from Tables 2,  3, and Table S1)

Mutation type	MLH1	MSH2	MSH6	PMS2	Total
Frameshift	30	29	25	2	86
Splice site	25	13	5	0	43
Nonsense	10	22	5	0	37
Exon del/dup	4	22	3	2	31
Missense	4	4	0	1	9
In frame del/dup	2	1	0	1	4
Rearrangement	0	1	0	0	1
Total	75	92	38	6	211

## Discussion

In this study, we report the MMR variants that have been identified in 333 consecutively collected families, from NSW, Australia over a period of almost 20 years, which have since been used for predictive testing. The MMR mutation spectrum in the present cohort is similar to that previously reported (Cohen and Leininger 2014). Mutations in *MLH1* and *MSH2* accounts for 80% of the total number of variants identified, whereas mutations in *MSH6* and *PMS2* accounts for almost 20%. The Amsterdam II criteria were fulfilled by 58% of the mutation positive families, which is in accordance with other studies (Syngal et al. 2000; Sjursen et al. 2010; Steinke et al. 2014).

The aim of this study was to investigate the variants used for predictive testing in order to establish whether they had been correctly classified and in accordance with the recently publish classification protocol suggested by the InSiGHT's collaboration on variant pathogenicity assessment. Sixty‐nine percent (146 out of 211 variants) were found to have a LOVD DB‐ID number and an InSiGHT classification. Of these, 141 (96.7%) were in accordance with our own interpretation as class 4 or 5. Five variants were defined as class 3 variants according to InSiGHT; whereas they were interpreted to be class 4 or 5 (Table 3) by us. Two of these variants were mutations in start codon of *MLH1* and *PMS2*, both of which are assigned pathogenic by several studies. Recent functional studies of *MLH1* c.A>G reveal that translation is mostly initiated at an in‐frame position 103 nucleotides downstream, but also at two ATG sequences downstream (Parsons et al. 2015). These two ATG sequences showed minimal protein expression (c.89ATG) or some expression (c.122ATG), but because it results in a reading frame shift these starting codons will lead to a truncated protein (Parsons et al. 2015). The protein product encoded by the in‐frame transcript initiating from position c.103 (lacking the first 34 amino acids) showed loss of in vitro mismatch repair activity comparable to known pathogenic mutations. Other nucleotide substitutions in *MLH1* start codon, c.2T>A (MLH1_00031) (Mangold et al. 2005) and c.2T>G (Bonadona et al. 2011; Canard et al. 2012) are reported as pathogenic because of abnormal IHC and MSI‐H tumors. The other start codon variant, *PMS2* c.1A>G, has previously been found in three index patients whose tumors showed loss of PMS2 staining, and where biallelic *PMS2* variants were found (Senter et al. 2008), as in our patient. Their patients had CRC in their twenties (Senter et al. 2008), while the index patient in our family had synchronous CRC and endometrial cancer at 55 years of age. In another study, monoallelic *PMS2* c.1A>G mutations were interpreted as pathogenic because of a lack of PMS2 protein staining in a patient with endometrial cancer diagnosed at 42 years of age (Borras et al. 2013). As described, there are several evidences for the pathogenicity of both *MLH1* and *PMS2* c.1A>G.

A third variant with discordance was *MLH1* c.988_990del p.[Ser295Argfs*21, Ile330del]. The variant has been shown by using a minigene assay to lead to partial exon 11 skipping (Tournier et al. 2008). It has been reported as a weak but reproducible factor contributing to exon 11 skipping, found in 5% of the transcripts. Blood samples suitable for RNA extraction were not available from patients carrying this *MLH1* variant, thus, the partial splicing effect has not been confirmed. Another functional study has shown that *MLH1* c.988_990del is associated with a reduction in protein expression and MMR activity and an alteration of subcellular localization of the MLH1 protein, (Raevaara et al. 2005) whereas interaction with PMS2 was comparable to WT. In addition to the functional assays implicating loss of MLH1 function, three have reported this in‐frame variant to be found in Amsterdam positive patients with tumors showing loss of MLH1 protein and MSI‐H to the LOVD/InSiGHT database (Desiree du Sart; MLH1_001631) (Southey et al. 2005; Jenkins et al. 2006). Therefore, according to the InSiGHT 5‐tiered classification system this variant should be classified as class 5.

The fourth discordant variant was *MSH6* c.3556+3_+13del, which we have confirmed by cDNA analyses to cause an aberrant transcript lacking exon 6. Thus, this variant should now be reclassified from class 3 to pathogenic class 5. The fifth discordant variant was *MSH2* c.2635‐3C>G. RNA analyses has not been performed by us, and we could not find any cDNA data in the literature either. It may cause aberrant splicing as indicated by the prediction programs, however, this must be confirmed by cDNA analyses. Thus, we agree with LOVD/InSiGHT that this is a class 3 variant, which not should be used for predictive testing. Taken together one out of five discordant variants has to be reclassified by us.

In addition to reported MMR mutations, we have presented 55 novel mutations (Table 2 and one novel in Table 3). The 64 mutations in Table 2 are classified as pathogenic (class 5) or probably pathogenic (class 4) as they are frameshift, nonsense, exon deletions or duplications, splice mutations, and a duplication of 66 nucleotides. Usually, nonsense and frameshift mutations result in premature termination codons which target transcripts for nonsense‐mediated mRNA decay. However, the effect of such mutations in the last exon cannot be determined conclusively. Three of the frameshift *MLH1* variants reported here are in the last exon (c.2114del, p.Pro705Leufs*78, c.2149del, p.Glu717Asnfs*66, and c.2196del, p.Lys732Asnfs*51). They alter the reading frame and change the last 51, 39 and 24 amino acids of exon 19, respectively. In addition, all three variants lead to extension of the protein by 25 amino acids. The C‐terminal end of MLH1 is important for dimerization to PMS2, and missense mutations of codon 749 and 750 are shown to be pathogenic due to the abolition of PMS2 binding (Kosinski et al. 2010). MLH1 needs to bind PMS2 to form a catalytically functional and correctly localized heterodimer, which is important in MMR. Therefore, these three *MLH1* frameshift mutations are interpreted to be class 4 variants and the families are offered predictive testing.

The nine novel splice mutations we report (Table 2) are in the highly conserved splice donor or acceptor sites (*n* = 5), or they were confirmed by RNA analyses to cause aberrant splicing (*n* = 3). One predicted splice mutation is located in the last nucleotide of exon 14 (*MLH1* c.1667G>A). We have interpreted *MLH1* c.1667G>A to be likely pathogenic because another substitution of the same nucleotide has been classified by InSiGHT to be pathogenic (*MLH1* c.1667G>T; MLH1_01162). In addition, *MLH1* c.1667G>C is published as likely pathogenic in ClinVar (accession RCV000164556.1). The predicted change at donor site one nucleotide downstream are almost equal for *MLH1* c.1667G>A (−43.4%) and c.1667G>C (−43.7%) compared to WT (c.1667G), whereas the predicted change for c.1667G>T is slightly higher (−55.7%). *MLH1* c.1667G>A was detected in one family fulfilling the Amsterdam criteria, and the proband and his brother were 49 and 36 years old at diagnosis of CRC, respectively.

One in‐frame duplication in *MSH2* (c.2051_2116dup; p.Val684_Val705dup) found in an Amsterdam positive family, has been interpreted as likely pathogenic because it lead to the insertion of 22 amino acids in the highly conserved ATP‐binding cassette domain of MSH2. Most probably this duplication disturbs the structure (http://www.uniprot.org/uniprot/P43246) of an important functional domain necessary for correct mismatch repair.

In our cohort, MSH2 variants are most frequent, both in number of variants (91 out of 211) and in number of families (147 out of 333). Thus, *MSH2* variants were the causative variant in almost half of our families. Most families have their own unique causative MMR variant. However, some variants were found in several families, whereof the most frequent were *MSH2* c.942+3A>T found in 11 families, and *MLH1* c.350C>T found in 8 families. The most frequent mutations found in our cohort are well known and have several public entries in the LOVD database. We have not identified any typical Australian founder mutations.

The most frequent mutation types in this study were frameshift, splice site, nonsense, and exon deletion/duplication mutations, accounting for 94% of the mutations. Exon deletion/duplication amounted to 15% of the mutations, and more than 2/3 (71%) of these were found in the *MSH2* gene. The spectrum of mutation types is similar to that found in other studies (Wijnen et al. 1998; Nilbert et al. 2009; Sjursen et al. 2010).

In conclusion, we found that most variants with an InSiGHT classification (141 out of 146) were in accordance with our classification. Five variants did not have the same classification, of which four can be reclassified by InSiGHT. In addition to already known MMR mutations, we have presented 55 novel pathogenic or putative pathogenic mutations.

## Conflict of Interest

The authors declare that there is no financial or relationships which might lead to a conflict of interest.

## Supporting information


**Table S1.** The 141 class 4 and 5 MMR variants identified which were found to have a LOVD DB‐ID number and an InSiGHT classification. Reference sequences used are MLH1: NM_000249.2, MSH2: NM_000251.2, MSH6: NM_000179.2, and PMS2: NM_000535.5.
