# Supplementary material for: Lynch syndrome mutation spectrum in New South Wales, Australia, including 55 novel mutations
Source: Mol Genet Genomic Med. 2016 Jan 11;4(2):223–31. doi: 10.1002/mgg3.198 (PMC4799874; doi:10.1002/mgg3.198)
Supplement: Supplementary file 1 — Table S1. The 141 class 4 and 5 MMR variants identified which were found to have a LOVD DB‐ID number and an InSiGHT classification. Reference sequences used are MLH1: NM_000249.2, MSH2: NM_000251.2, MSH6: NM_000179.2, and PMS2: NM_000535.5. [file MGG3-4-223-s001.docx]

| **Gene** | **Nucleotide change *** | **Consequence of mutation** | **LOVD ID** | **No of families** |
| --- | --- | --- | --- | --- |
| **MLH1** | c.1_2271del | Whole gene deletion  (c.198-?_*193_?del) | MLH1_00945 | 2 |
|  | c.9delC | Frameshift, p.(Phe3Leufs*14) | MLH1_00966 | 1 |
|  | c.67G>T | Nonsense, also partial splicing aberration: p.[Glu23*, Glu23_Cys39del] | MLH1_00045 | 1 |
|  | c.116G>A | Splice site, p.?, last base of exon | MLH1_00026  MLH1_01456¤ | 3 |
|  | c.116+5G>C | Splice site, p.Cys39Trpfs*11 | MLH1_01083¤ | 2 |
|  | c.131C>T | Missense, p.(Ser44Phe) | MLH1_00071 | 1 |
|  | c.208-3C>G | Splice site, p.(Lys70_Glu102del) | MLH1_01263 | 1 |
|  | c.306+1G>A | Splice site, p.? | MLH1_00170 | 1 |
|  | c.307-1G>C | Splice site, p.? | MLH1_01297¤ | 1 |
|  | c.307_453del | Exon deletion, exon 4-5;  (c.307-?_453+?del) | MLH1_01589¤ | 1 |
|  | c.350C>T | Missense, p.(Thr117Met) | MLH1_00204¤ | 8 |
|  | c.380+1G>A | Splice site, p.? | MLH1_00213¤ | 2 |
|  | c.404_407delTGAA | Frameshift, p.(Leu135Glnfs*24); | MLH1_01459¤ | 1 |
|  | c.454_545del c.454-?_545+?del | Exon deletion, exon 6; p.(Glu153Phefs*8) | MLH1_01604 | 2 |
|  | c.554T>G | Missense, p.(Val185Gly) | MLH1_00229 MLH1_01593¤ | 4 |
|  | c.574_588+2del | Deletion, Interrupts canonical donor splice site, p.? | MLH1_01317 | 1 |
|  | c.588delA | Frameshift, p.(Lys196Asnfs*6) | MLH1_00260¤ | 1 |
|  | c.588+1G>T | Splice site, r.546_588del, p.(Arg182Serfs*6) | MLH1_01321¤ | 2 |
|  | c.672delT | Frameshift, p.(Ser225Valfs*4) | MLH1_01336  MLH1_01461¤ | 1 |
|  | c.677G>A | Splice site, p.(Gln197Argfs*8), last base of exon | MLH1_00283 | 2 |
|  | c.677+1G>T | Splice site, p.? | MLH1_01338 | 1 |
|  | c.678-1G>T a) | Splice site mutation, p.(Glu227Serfs*42) | MLH1_01344¤ | 1 |
|  | c.678-2A>G | Splice site, p.? | MLH1_00906 | 1 |
|  | c.678-9_693del (c.678-11del25) b) | Deletion, Interrupts canonical donor splice site, p.? | MLH1_01342  MLH1_01474¤ | 1 |
|  | c.790+1G>A | Splice site, p.? | MLH1_00340 | 1 |
|  | c.884G>A | Splice site, p.(His264Leufs*2), last base of exon | MLH1_01475¤ | 1 |
|  | c.1163_1164insGAAT | Frameshift, p.(Arg389Asnfs*7) | MLH1_01051¤ | 1 |
|  | c.1190delT | Frameshift, p.(Leu397Argfs*4) | MLH1_01088 | 1 |
|  | c.1380_1381del c) | Frameshift, p.(Lys461Glufs*17) | MLH1_00844 | 1 |
|  | c.1409+1G>A | Splice site, (Lys461Glufs*17) | MLH1_01108 | 1 |
|  | c.1459C>T | Nonsense, p.(Arg487*) | MLH1_00511 | 2 |
|  | c.1464_1468delGGAAA | Frameshift, p.(Lys488Asnfs*13) | MLH1_01464¤ | 4 |
|  | c.1489dup (c.1483_1484insC) ^b)^ | Frameshift, p.(Arg497Profs*6) | MLH1_00513¤ | 1 |
|  | c.1534G>T | Nonsense, p.(Glu512*) | MLH1_00519 | 1 |
|  | c.1554dupT ^d)^ | Frameshift, p.(Glu519*) | MLH1_01578 | 2 |
|  | c.1559-1G>C | Splice site, p.? | MLH1_00547¤ | 1 |
|  | c.1559-2A>G | Splice site, skipping of exon 14-15 | MLH1_00536 | 1 |
|  | c.1574T>A | Nonsense, p.(Leu525*) | MLH1_01466¤ | 1 |
|  | c.1640T>A | Nonsense, p.(Leu547*) | MLH1_00568¤ | 1 |
|  | c.1668-1G>A | Splice site, p.? | MLH1_01166¤ | 2 |
|  | c.1683C>G | Nonsense, p.(Tyr561*) | MLH1_01172¤ | 2 |
|  | c.1731G>A | Splice site, p.(Ser556Argfs*14), last base in exon | MLH1_00598  MLH1_01468¤ | 5 |
|  | c.1732_2271del | Exon deletion, exon 16-19  (c.1732-?_*193_?del) | MLH1_01183 | 5 |
|  | c.1758delC | Frameshift, p.(Met587Cysfs*4) | MLH1_01191 | 1 |
|  | c.1852_1854delAAG | In frame deletion, r.1852_1854del, p.(Lys618del) | MLH1_00652 | 4 |
|  | c.1866delT | Frameshift, p.(Ala623Glnfs*14) | MLH1_01469¤ | 1 |
|  | c.1975C>T | Nonsense, p.(Arg659*), r.[1897_1989del, 1975c>u], p.[Glu633_Glu663del, Arg659*] | MLH1_00688 | 1 |
|  | c.1975_1976delCG | Frameshift, p.(Arg659Thrfs*4) | MLH1_00204¤ | 4 |
|  | c.1989G>A | Splice site, p.?, last base in exon | MLH1_01236 | 1 |
|  | c.1989+1G>A | Splice site, p.? | MLH1_00727¤ | 2 |
|  | c.2006-2A>G | Splice site, p.? | MSH2_01078 | 4 |
|  | c.2006_2010del | Frameshift, p.(Glu669Glyfs*4) | MLH1_01247 | 1 |
|  | c.2252_2253delAA e) | Frameshift, p.(Lys751Serfs*3) | MLH1_00794¤ | 1 |
| **MSH2** | c.1-?_211+?del | Exon deletion; exon 1 | MSH2_00860 | 2 |
|  | c.1_366del | Exon deletion, exon 1-2 | MSH2_00862 | 3 |
|  | c.1_792del ^a)^ | Exon deletion, exon 1-4 | MSH2_00864 | 1 |
|  | c.1_1276del | Exon deletion; exon 1-7 | MSH2_00856 | 3 |
|  | c.1_2805del ^b)^ | Whole gene deletion | MSH2_00043 | 3 |
|  | c.142G>T | Nonsense, p.(Glu48*) | MSH2_00955 | 2 |
|  | c.163delC | Frameshift, p.(Arg55Glyfs*9) | MSH2_00009 | 1 |
|  | c.181C>T | Nonsense, (p.Gln61*) | MSH2_00015 | 1 |
|  | c.186_187dupGG | Frameshift, p.(Val63Glyfs*2) | MSH2_01048 | 4 |
|  | c.212-1G>A | Splice site, p.? | MSH2_01110 | 1 |
|  | c.212_1076del | Exon deletion; exon 2-6, p.(Gly71Aspfs*2) | MSH2_00088 | 2 |
|  | c.229_230delAG ^b)^ | Frameshift, p.(Ser77Cysfs*4) | MSH2_00100 | 1 |
|  | c.263_264del | Frameshift, p.(Phe88Cysfs*11) | MSH2_00765 | 1 |
|  | c.289C>T | Nonsense, p.(Gln97*) | MSH2_00083 | 1 |
|  | c.367_645del | Exon deletion, exon 3 | MSH2_00115 | 2 |
|  | c.388_389delCA | Frameshift, p.(Gln130Valfs*2) | MSH2_00136 | 1 |
|  | c.484G>A | Missense, p.(Gly162Arg) | MSH2_00152 | 1 |
|  | c.508C>T | Nonsense, p.(Gln170*) | MSH2_00139 | 1 |
|  | c.547C>T | Nonsense, p.(Gln183*) | MSH2_01239 | 1 |
|  | c.645+1G>A | Splice site, p.(Ala123_Gln215del ) | MSH2_01251 | 2 |
|  | c.646_792del | Exon deletion, exon 4 | MSH2_01256 | 1 |
|  | c.646_2674del | Exon deletion, exon 4-16 | MSH2_01255 | 1 |
|  | c.679delA | Frameshift, p.(Arg227Glufs*19) | MSH2_01409 | 1 |
|  | c.687delA ^f)^ | Frameshift, p.(Ala230Leufs*16) | MSH2_00191 | 1 |
|  | c.811_814delTCTG | Frameshift, p.(Ser271Argfs*2) | MSH2_01285 | 1 |
|  | c.892C>T | Nonsense, p.(Gln298*) | MSH2_00234 | 3 |
|  | c.942+3A>T | Splice site, r.793_942del | MSH2_00260 | 11 |
|  | c.958dupA | Frameshift, p.(Thr320Asnfs*13) | MSH2_00285 | 1 |
|  | c.1009C>T | Nonsense, p.(Gln337*) | MSH2_00271¤ | 6 |
|  | c.1034G>A | Nonsense, p.(Trp345* ) | MSH2_00867 | 1 |
|  | c.1076+1G>A | Splice site, r.943_1076del | MSH2_00291 | 1 |
|  | c.1077-2A>T | Splice site, p.? | MSH2_00879 | 4 |
|  | c.1077_1276dup | Exon duplication, exon 7 | MSH2_00873 | 1 |
|  | c.1147C>T | Nonsense, p.(Arg383*) | MSH2_00310 | 3 |
|  | c.1165C>T | Nonsense, p.(Arg389*) | MSH2_00311 | 2 |
|  | c.1204C>T | Nonsense, p.(Gln402*) | MSH2_00897 | 2 |
|  | c.1216C>T | Nonsense, p.Arg406* | MSH2_00312 | 3 |
|  | c.1277_1386del | Exon deletion, exon 8, p.(Lys427Glyfs*4) | MSH2_00378 | 3 |
|  | c.1311_1334delins1338_1361inv | Rearrangement, p.? | MSH2_00930¤ | 1 |
|  | c.1340_1341insGG | Frameshift, p.(Phe447Leufs*8) | MSH2_00384 | 1 |
|  | c.1387_1662del | Exon deletion, exon 9-10 | MSH2_00946 | 1 |
|  | c.1387_2458del ^b)^ | Exon deletion, exon 9-14 | MSH2_00947 | 1 |
|  | c.1408delG | Frameshift, p.(Val470*) | MSH2_00952¤ | 2 |
|  | c.1552_1553delCA | Frameshift, p.(Gln518Valfs*10) | MSH2_00435 | 2 |
|  | c.1578delC | Frameshift: p.(Glu530Lysfs*13) | MSH2_00437 | 1 |
|  | c.1662_1759del | Exon deletion, exon 11 | MSH2_01000 | 1 |
|  | c.1705_1706delGA | Frame shift, p.(Glu569Ilefs*2) | MSH2_00476 | 2 |
|  | c.1720C>T ^b)^ | Nonsense, p.(Gln574*) | MSH2_01024 | 1 |
|  | c.1838dupA | Frameshift, p.(Asn613Lysfs*31) | MSH2_01488 | 2 |
|  | c.1853delC | Frameshift, p.(Pro618Hisfs*17) | MSH2_01044 | 1 |
|  | c.1865C>T | Missense, p.(Pro622Leu) | MSH2_00547 | 1 |
|  | c.1889_1892delGAAG | Frameshift, p.(Gly630Glufs*4) | MSH2_01051 | 3 |
|  | c.1906G>C ^g)^ | Missense, p.(Ala636Pro) | MSH2_00508 | 1 |
|  | c.1968G>C | Nonsense, p.(Tyr656*) | MSH2_00557¤ | 1 |
|  | c.2005+2T>C | Splice site, p.? | MSH2_00565 | 1 |
|  | c.2006-2A>G | Splice site, p.? | MSH2_01078 | 2 |
|  | c.2038C>T ^a)^ | Nonsense, p.(Arg680*) | MSH2_00580 | 1 |
|  | c.2131C>T | Nonsense, p.Arg711* | MSH2_00581 | 1 |
|  | c.2135dupT | Frameshift, p.(Pro670Leufs*7) | MSH2_01111 | 1 |
|  | c.2228C>G | Nonsense, p.(Ser743*) | MSH2_00646 | 2 |
|  | c.2362dupA | Frameshift, p.(Thr788Asnfs*11) | MSH2_01151 | 1 |
|  | c.2502_2508delTAATTTC | Frameshift, p.(Asn835Leufs*4) | MSH2_00665¤ | 6 |
|  | c.2634+5G>C | Splice site, r.2459_2634del | MSH2_01181 | 1 |
| **MSH6** | c.1_457del | Exon deletion; exon 1-2 | MSH6_00001# | 1 |
|  | c.458-?_627+?del | Exon deletion; exon 3 | MSH6_00336# | 6 |
|  | c.710delG | Frameshift, p.(Gly237Aspfs*9) | MSH6_00702# | 1 |
|  | c.2150_2153del | Frameshift, p.(Val717Alafs*18) | MSH6_00175# | 3 |
|  | c.2348_2349del | Frameshift, p.(Cys783*) | MSH6_00442# | 1 |
|  | c.2535dup | Frameshift, p.(Glu846*) | MSH6_00701# | 1 |
|  | c.2731C>T | Nonsense, p.(Arg911*) | MSH6_00071# | 1 |
|  | c.2764C>T | Nonsense, p.(Arg922*) | MSH6_00699¤ | 3 |
|  | c.2765delG ^b)^ | Frameshift, p.(Arg922Glnfs*23) | MSH6_00703# | 1 |
|  | c.2976delA | Frameshift, p.(Glu993Asnfs*4) | MSH6_00709# | 1 |
|  | c.3020G>A | Nonsense, p.(Trp1007*) | MSH6_00472 | 1 |
|  | c.3172+1G>T | Splice site, p.? | MSH6_00705# | 2 |
|  | c.3173_3556del | Exon deletion, exon 5-6 | MSH6_00482# | 1 |
|  | c.3202C>T | Nonsense, p.(Arg1068*) | MSH6_00487# | 2 |
|  | c.3261del | Frameshift, p.(Phe1088Serfs*2) | MSH6_00203# | 3 |
|  | c.3261dupC | Frameshift, p.(Phe1088Leufs*5) | MSH6_00201# | 4 |
|  | c.3268_3274del | Frameshift, p.(Glu1090Lysfs*23) | MSH6_00706# | 1 |
|  | c.3312delT | Frameshift, p.(Phe1104Leufs*11) | MSH6_00708# | 2 |
|  | c.3939_3957dup ^b)^ | Frameshift, p.(Ala1320Serfs*5) | MSH6_00674 | 1 |
|  | c.3439-1G>T | Splice site, p.? | MSH6_00713# | 2 |
|  | c.3514dupA | Frameshift, p.(Arg1172Lysfs*5) | MSH6_00700¤ | 1 |
|  | c.3725_3737del ^h^ | Frameshift, p.(Arg1242Glnfs*7) | MSH6_00715 | 1 |
|  | c.3920_3927dup ^i)^ | Frameshift, p.(Glu1310Ilefs*20) | MSH6_00332 | 1 |
| **PMS2** | c.736_741del6ins11 | Frameshift, p.(Pro246Cysfs*3) | PMS2_00187# | 3 |
|  | c.904_1144del | Exon deletion, exon 9-10 | PMS2_00195# | 2 |

**Supplementary Table:** The 141 class 4 and 5 MMR variants identified which were found to have a LOVD DB-ID number and an InSiGHT classification. Reference sequences used are MLH1: NM_000249.2, MSH2: NM_000251.2, MSH6: NM_000179.2 and PMS2: NM_000535.5

¤ Submitted by R. Scott

# Reported in B. Talseth-Palmer et al, 2010

* Mutation originally identified by:

a)Queensland Health Pathology Service, Molecular Genetics Laboratory.

b) Victorian Clinical Genetics Service, Molecular Genetics Laboratory.

c) NW Thames Regional Genetics Service, Medical Genetics Laboratory.

d) Queensland Clinical Genetics Service, Royal Children’s Hospital.

e) Institute of Human Genetics, Newcastle Upon Tyne.

f) Northern Ireland Regional Genetics Centre, Belfast City Hospital.

g) Molecular Genetics Unit, Princess Margaret Hospital, Western Australia.

h) Flinders Medical Centre, Genetic Pathology Laboratory, South Australia

i) Klinische Genetica, VU Medisch Centrum, Amsterdam, The Netherlands.
